# Supplementary material for: Low Energy Shock Wave Therapy Inhibits Inflammatory Molecules and Suppresses Prostatic Pain and Hypersensitivity in a Capsaicin Induced Prostatitis Model in Rats
Source: Int J Mol Sci. 2019 Sep 26;20(19):4777. doi: 10.3390/ijms20194777 (PMC6801724; doi:10.3390/ijms20194777)
Supplement: Supplementary file 1 [file ijms-20-04777-s001.zip › supplementary material/IJMS suppl 1.pdf]

Supplement Table. 1

General characteristics of the experimental animals, n=8 in each group.

	Mean±SEM				
	Sham control	Capsaicin (Cap.)	Cap.+LESW 100	Cap.+LESW 200	Cap.+LESW 300
**Day 3**					
Body wt (gm), day 0	301.82±8.49	320.96±9.94	345.94±15.81	343.23±12.14	343.51±14.66
Body wt (gm), day 3	306.55±8.72	325.57±9.74	349.00±14.17	344.62±11.97	344.70±13.86
Difference of body wt (gm)	4.730±1.65	4.61±1.64	3.06±2.88	1.39±1.26	1.20±2.13
Prostate wt (mg)	330±2.34	350±2.35	442±3.11	419±3.81	436±3.11
Prostate wt/Body wt (mg/g)	1.1±0.06	1.1±0.1	1.2±0.1	1.2±0.1	1.2±0.1
**Day 7**					
Body wt (gm), day 0	322.08±19.32	322.78±12.34	309.33±14.25	311.90±9.06	314.84±12.73
Body wt (gm), day 7	337.17±18.06	343.39±11.29	333.87±12.96	328.70±8.23	333.40±11.52
Difference of body wt (gm)	15.08±3.19	20.61±3.02	24.53±3.94	16.80±3.21	18.56±3.53
Prostate wt (mg)	370±3.1	390±2.68	360±2.1	370±2.5	340±1.91
Prostate wt/Body wt (mg/g)	1.1±0.1	1.2±0.08	1.1±0.08	1.13±0.06	1.03±0.04

The table show general characteristics of the experimental animals. Data presented as the means ± SE of 8 rat/group.
