# Supplementary material for: Low Energy Shock Wave Therapy Inhibits Inflammatory Molecules and Suppresses Prostatic Pain and Hypersensitivity in a Capsaicin Induced Prostatitis Model in Rats
Source: Int J Mol Sci. 2019 Sep 26;20(19):4777. doi: 10.3390/ijms20194777 (PMC6801724; doi:10.3390/ijms20194777)
Supplement: Supplementary file 1 [file ijms-20-04777-s001.zip › supplementary material/The Effect of ESWT on Capsaicin original immunoblot.pdf]

Low energy shock wave therapy inhibits inflammatory molecules and suppresses prostatic pain and hypersensitivity in capsaicin induced prostatitis model in rats

Hung-Jen Wang[1,2], Pradeep Tyagi[3], Yu-Ming Chen[2], Michael B. Chancellor[4], Yao-Chi Chuang[1,2*]

Western blot of prostate tissue extract expression at 3-day

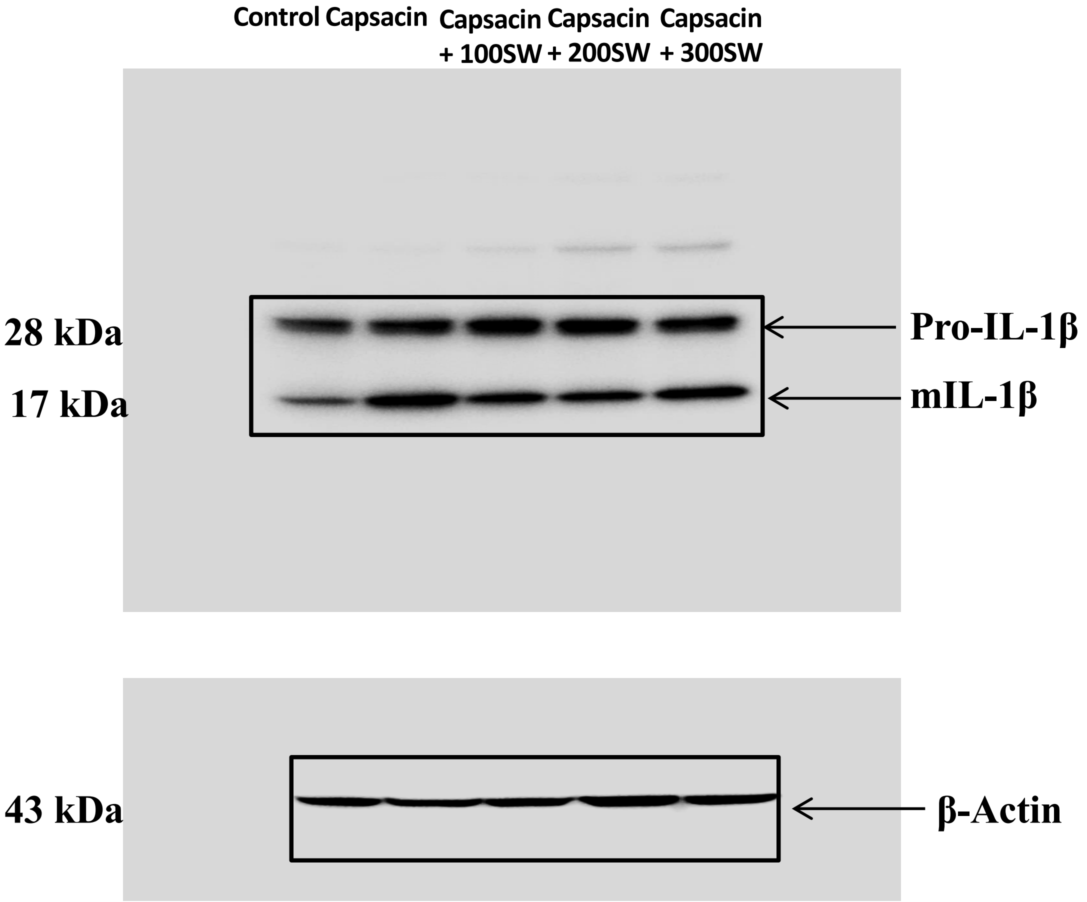

Supplementary figure S1a. Original blots for figure 4

Western blot of prostate tissue extract expression at 3-day

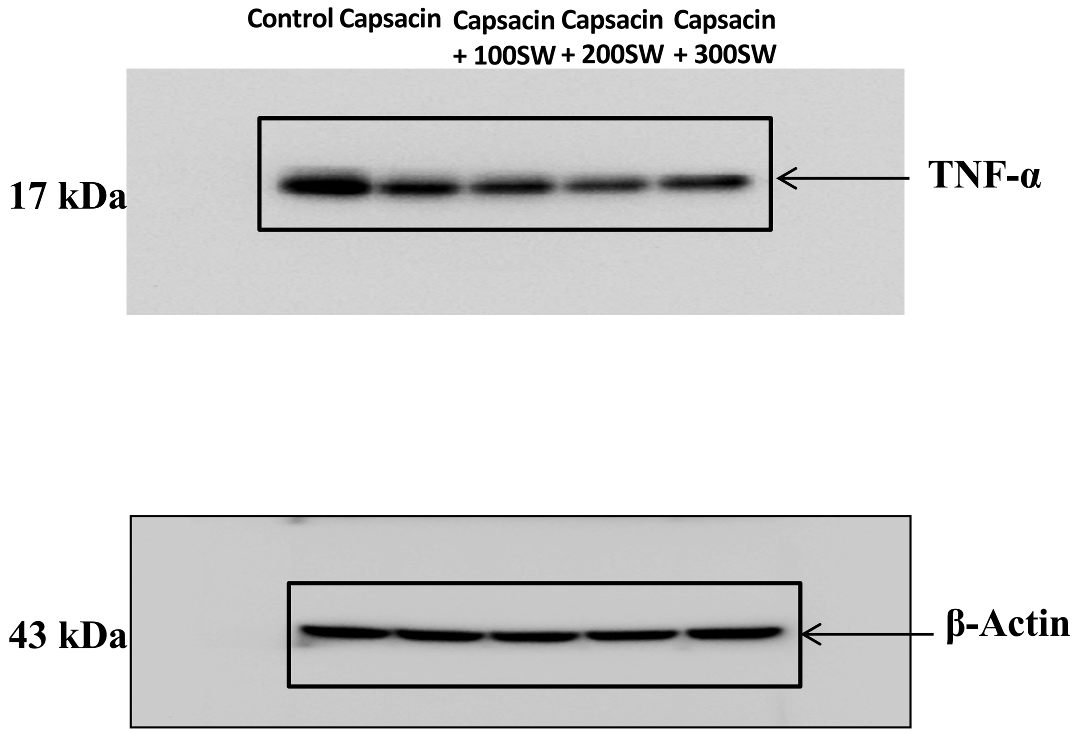

Supplementary figure S1b. Original blots for figure 4

Western blot of prostate tissue extract expression at 3-day

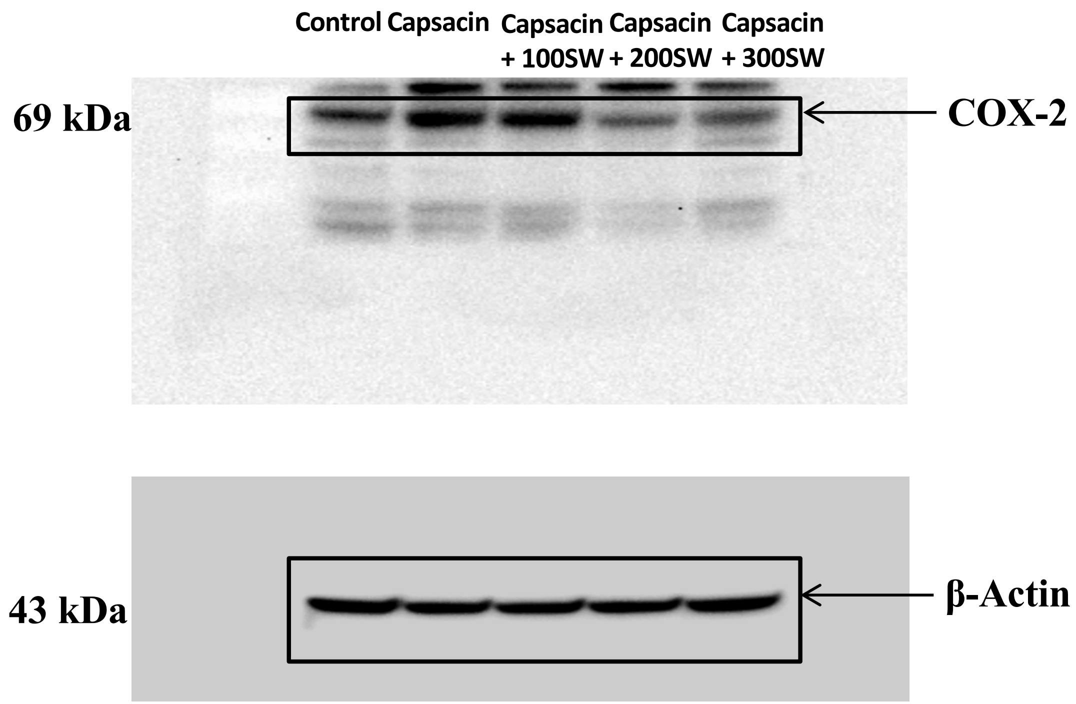

Supplementary figure S1c. Original blots for figure 4

Western blot of prostate tissue extract expression at 3-day

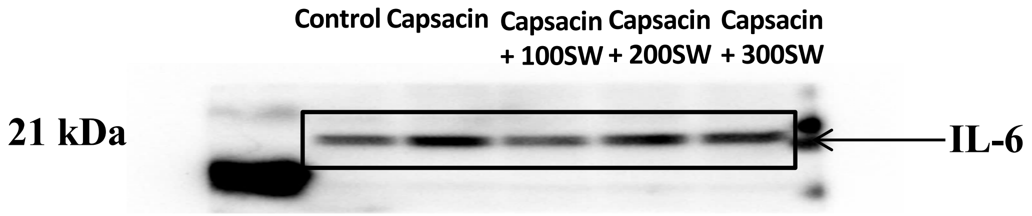

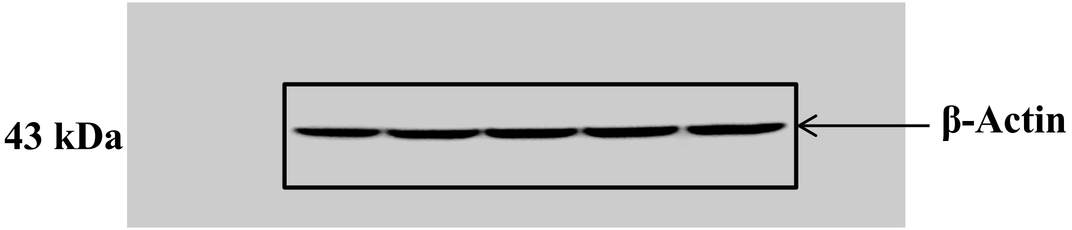

Supplementary figure S1d. Original blots for figure 4

Western blot of prostate tissue extract expression at 3-day

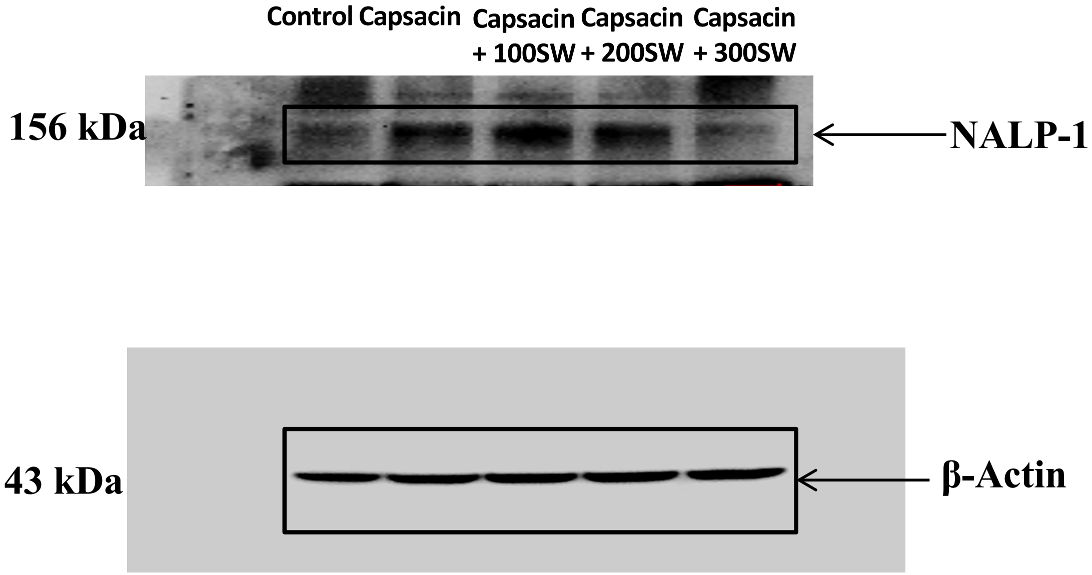

Supplementary figure S1e. Original blots for figure 4

Western blot of prostate tissue extract expression at 3-day

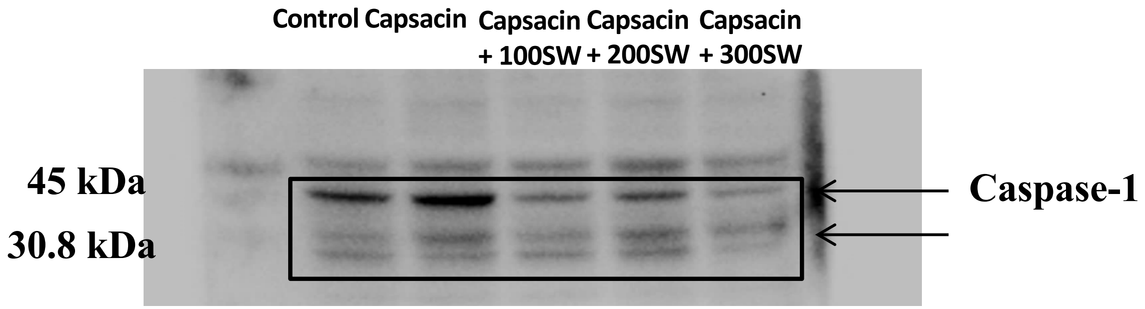

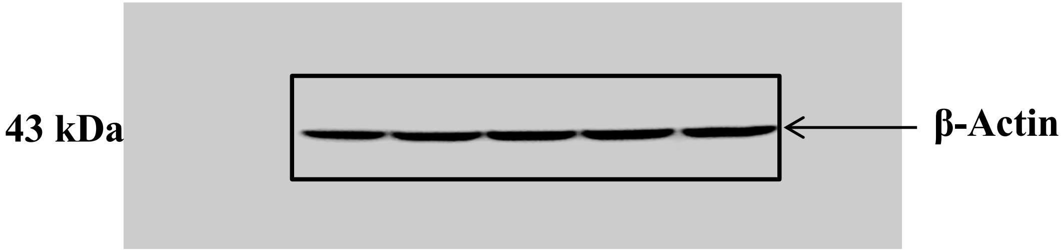

Supplementary figure S1f. Original blots for figure 4

Western blot of prostate tissue extract expression at 3-day

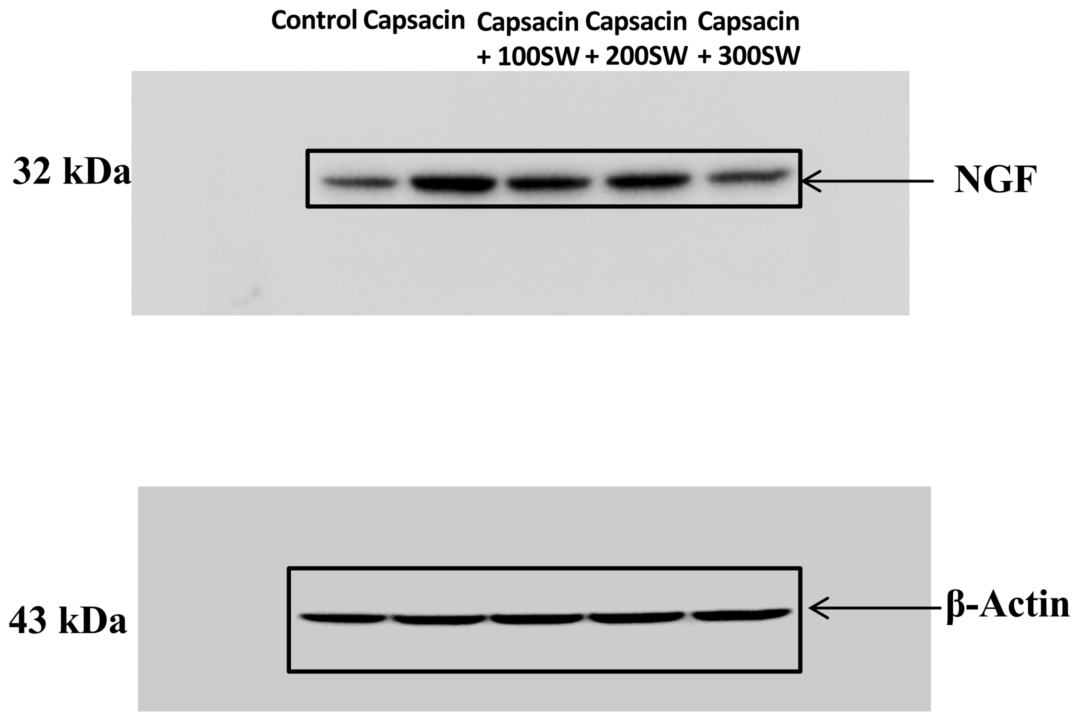

Supplementary figure S1g. Original blots for figure 4

Western blot of prostate tissue extract expression at 7-day

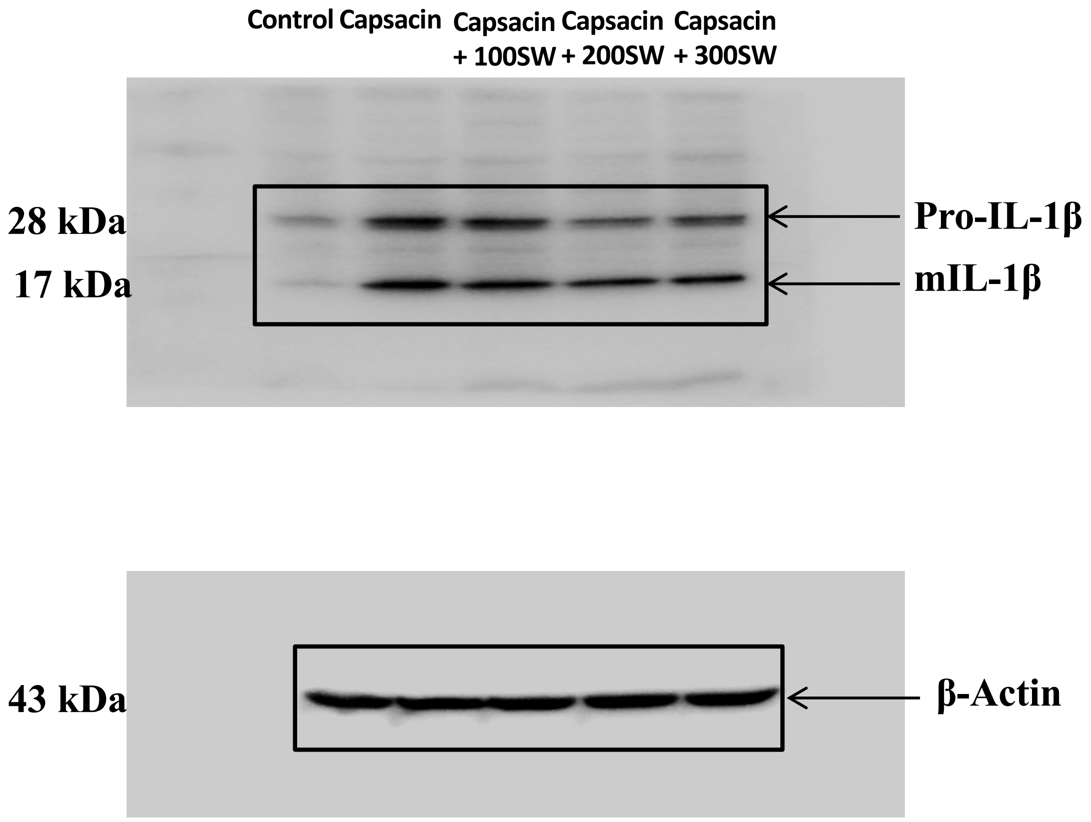

Supplementary figure S2a. Original blots for figure 5

Western blot of prostate tissue extract expression at 7-day

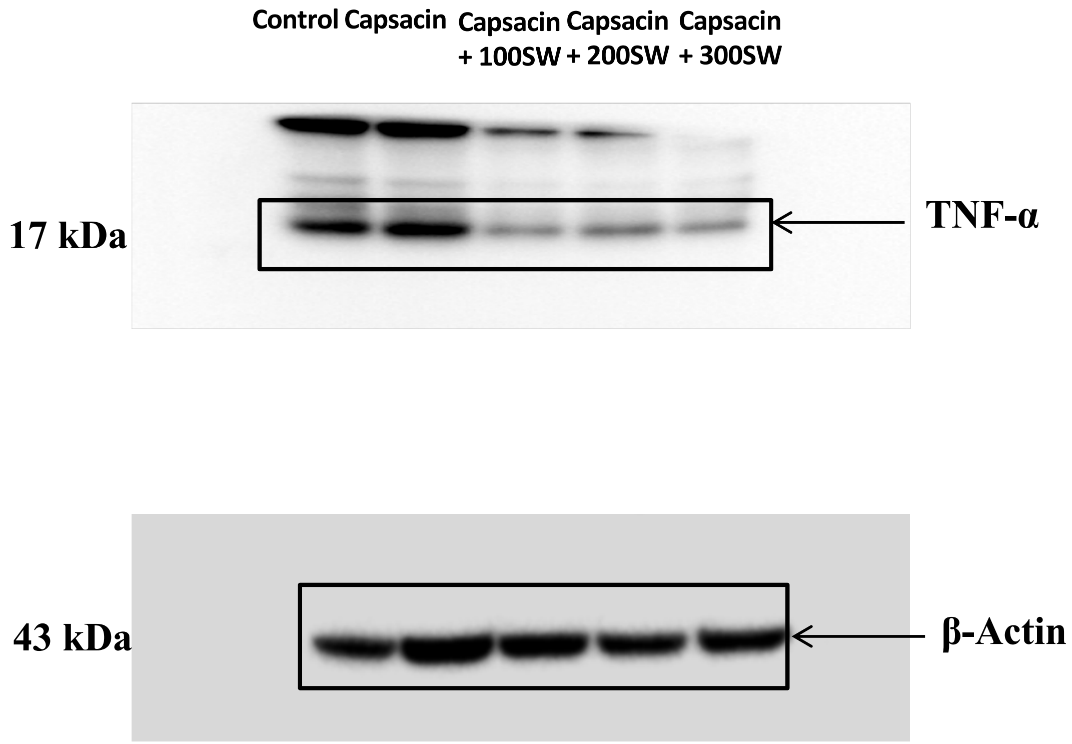

Supplementary figure S2b. Original blots for figure 5

Western blot of prostate tissue extract expression at 7-day

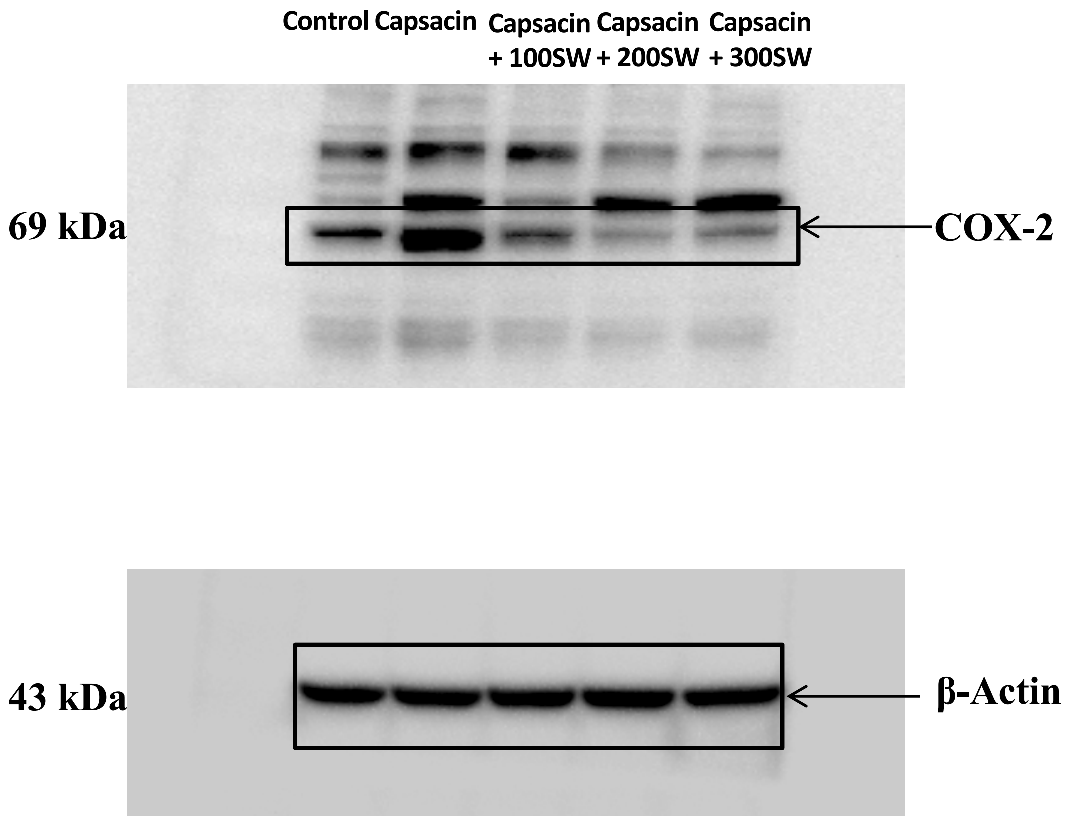

Supplementary figure S2c. Original blots for figure 5

Western blot of prostate tissue extract expression at 7-day

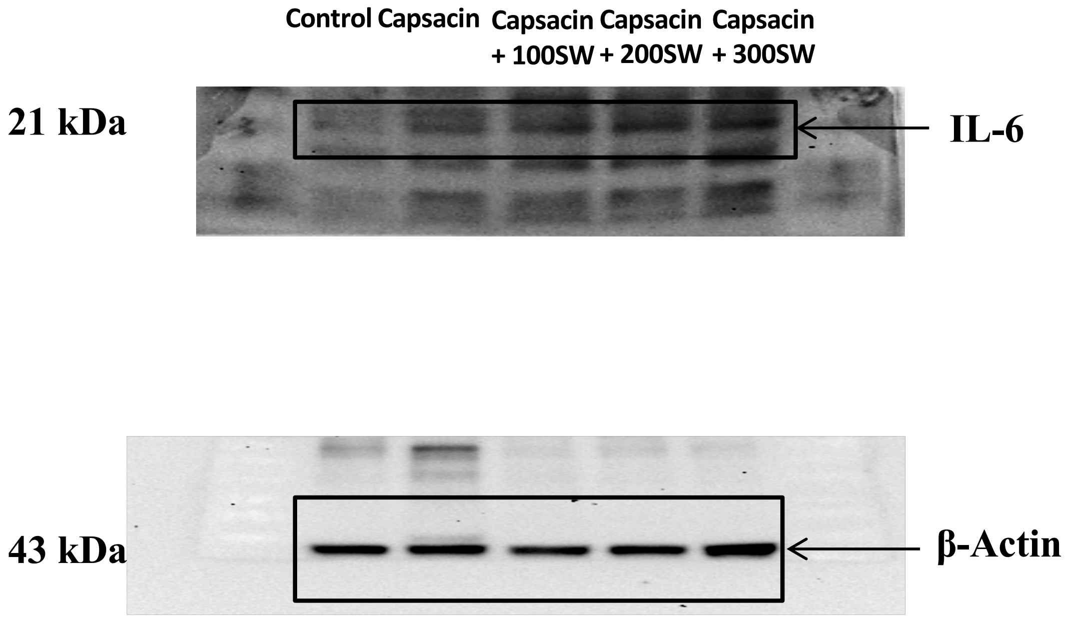

Supplementary figure S2d. Original blots for figure 5

Western blot of prostate tissue extract expression at 7-day

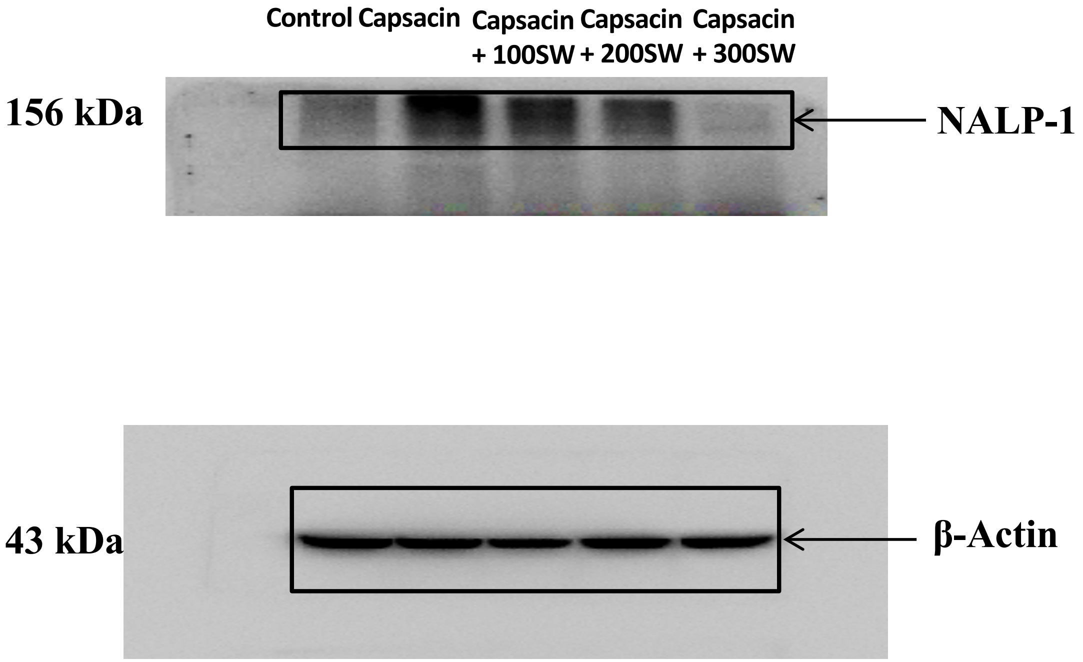

Supplementary figure S2e. Original blots for figure 5

Western blot of prostate tissue extract expression at 7-day

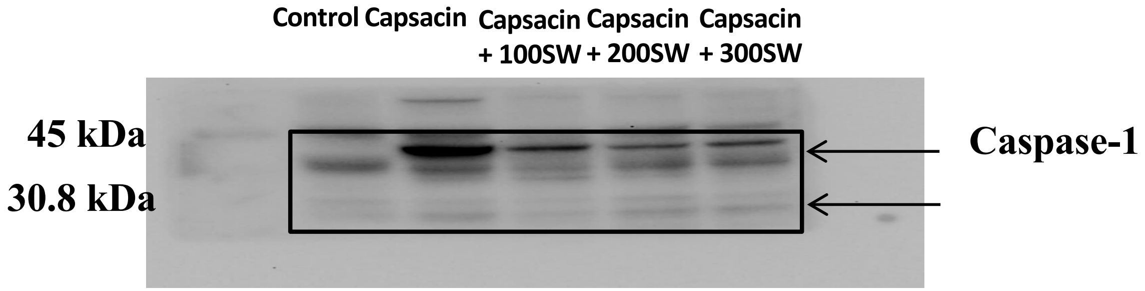

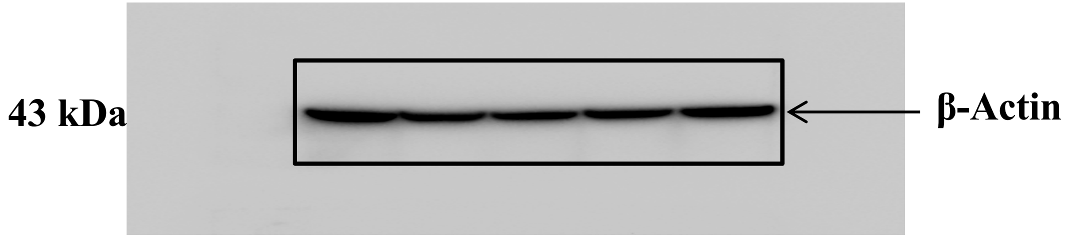

Supplementary figure S2f. Original blots for figure 5

Western blot of prostate tissue extract expression at 7-day

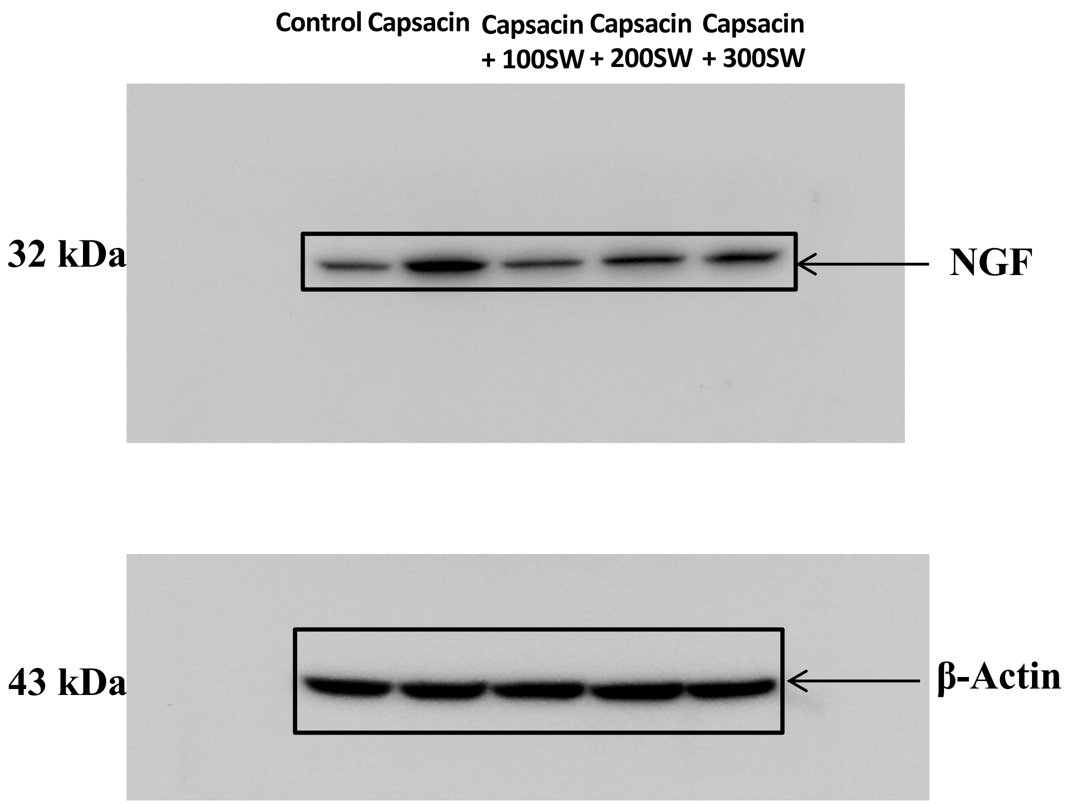

Supplementary figure S2g. Original blots for figure 5
